# Three New Butenolides from the Fungus *Aspergillus* sp. CBS-P-2

**DOI:** 10.3390/molecules21101361

**Published:** 2016-10-13

**Authors:** Xiao An, Yuehu Pei, Shaofei Chen, Shengge Li, Xiaolong Hu, Gang Chen, Bin Lin, Haifeng Wang

**Affiliations:** 1 School of Traditional Chinese Materia Medica, Shenyang Pharmaceutical University, Shenyang 110016, China; anxiao8520193@163.com (X.A.); peiyueh@vip.163.com (Y.P.); csf19920125@126.com (S.C.); lsg1215@sina.com (S.L.); huxiaolongbang@163.com (X.H.); chengang1152001@163.com (G.C.); 2 Key Laboratory of Structure-Based Drug Design and Discovery, Ministry of Education, Shenyang Pharmaceutical University, Shenyang 110016, China; randybinlin@sina.com; 3 Department of Medicinal Chemistry, School of Pharmaceutical Engineering, Shenyang Pharmaceutical University, Shenyang 110016, China

**Keywords:** *Aspergillus* sp*.*, butenolides, antioxidation, cytotoxicity, antimicrobial activity

## Abstract

Three new butenolides aspernolides H–J (**1**–**3**) together with seven known ones (**4**–**10**) were isolated from the fungus *Aspergillus* sp. CBS-P-2. Their chemical structures were established on the basis of 1D- and 2D-NMR spectroscopic data, HR-ESI-MS analysis, and their absolute configuration were determined by circular dichroism (CD) analysis. All the compounds were evaluated for the antioxidant effects by DPPH and ABTS methods, the antitumor activities against four human tumor cell lines (HL-60, ASPC1, HCT-116 and PC-3) and antimicrobial activities. Compounds **4**–**10** showed significant activity against DPPH (IC_50_ = 15.9–34.3 μM) and compounds **1**–**10** exhibited significant ABTS free radical scavenging activity (IC_50_ = 2.8–33.1 μM). Compounds **2**, **5** and **11** showed potent cytotoxic activities against HL-60 cell lines with IC_50_ values of 39.4, 13.2 and 16.3 μM, respectively. Compound **10** showed good antimicrobial activity against *Staphylococcus aureus* with minimum inhibitory concentration (MIC) of 21.3 μM.

## 1. Introduction

Recently, attention has been focused on the biology and chemistry of the microorganisms from extreme environments [1]. These microorganisms have proved to be one of the most important and underexplored resources for structurally novel and biologically active natural products [2,3]. *Aspergillus* species from diverse extreme origins (e.g., salt soil, plants, marine organisms and wetland) are important fungi that biosynthesize structurally diverse and pharmaceutically important natural products, such as alkaloids [4], polyketides [5], terpenes [6], and peptides [7], with intriguing biological properties. Butyrolactones and aspernolides, with a basal skeleton characterized by a five-membered lactone bearing two aromatic rings [8,9], mainly produced by *Aspergillus* sp. exhibited a wide range of activities, such as selectively inhibitory activities against CDK2 [10], α-glucuronidase [11] and cytotoxic [12], antioxidant [13], anti-inflammatory [14] activities. 

**Figure 1:**
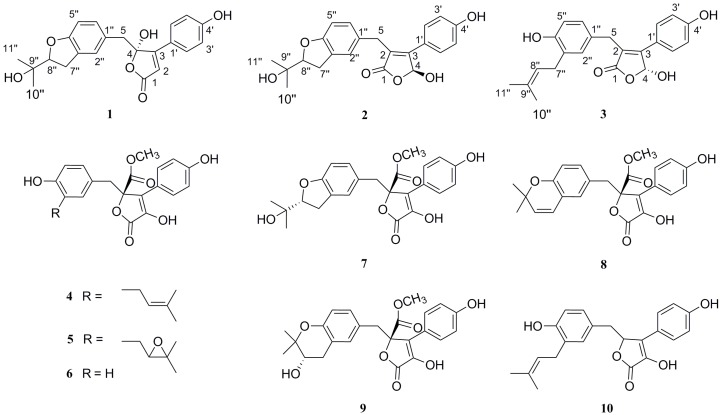
The structures of compounds **1**–**10**.

As a part of our continuing interest in exploring bioactive metabolites from fungal source, three new aspernolides (**1**–**3**) along with seven known compounds (**4**–**10**) (Figure 1) were isolated from the fungus *Aspergillus* sp. CBS-P-2, which was isolated from the volcanic soil collected in Changbai Mountain, Jilin, P.R. China. The structures of the isolated compounds were established on the basis of 1D, 2D NMR and HR-ESI-MS spectral data. In addition, the antioxidant effects, the cytotoxic properties against four human tumor cell lines [human leukaemia cancer (HL-60), human pancreatic cancer (ASPC1), human colon cell (HCT-116) and human prostatic cancer (PC-3)] and the antimicrobial activities of compounds **1**–**10** have been evaluated.

## 2. Results and Discussion

The fermentation broth of the fungal strain *Aspergillus* sp. CBS-P-2 was extracted with EtOAc and then concentrated under reduced pressure to give an extract. The EtOAc extract was subjected to various column chromatography protocols to afford compounds **1**–**10**. These new structures were identified by spectroscopic analyses and physicochemical properties, while the known analogues were identified as butyrolactone I (**4**) [15], butyrolactone ΙΙΙ (**5**) [15], butyrolactone ΙΙ (**6**) [15], butyrolactone IV (**7**) [15], aspernolide E (**8**) [16], butyrolactone V (**9**) [8] and 3-hydroxy-5-[[4-hydroxy-3-(3-methyl-2-buten-1-yl)phenyl]methyl]-4-(4-hydroxyphenyl)-2(5*H*)-furanone (**10**) [17] by comparison of their spectroscopic data and specific rotations with those in the literature.

**Figure 2:**
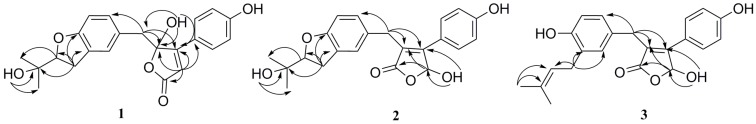
The key HMBC correlations of compounds **1**–**10**.

Compound **1** was isolated as a light yellowish gum. The molecular formula of **1** was determined to be C_22_H_22_O_6_ by positive mode HR-ESI-MS data at *m*/*z* 405.1300 [M + Na]^+^ (calcd. for C_22_H_22_O_6_Na, 405.1314), indicating 12 degrees of unsaturation. The ^1^H-NMR spectrum of **1** (Table 1) showed characteristic signals attributable to an olefinic proton singlet δ_H_ 6.23 (1H, s, H-2), a benzyl moiety with an 1, 2, 4-trisubstituted aromatic system [δ_H_ 6.48 (1H, brs, H-2′′), δ_H_ 6.46 (1H, d, *J* = 8.4 Hz, H-5′′), δ_H_ 6.54 (1H, brd, *J* = 8.4 Hz, H-6′′)] and a para-disubstituted benzene ring [δ_H_ 7.74 (2H, d, *J* = 8.4 Hz, H-2′/H-6′), 6.89 (2H, d, *J* = 8.4 Hz, H-3′/5′)]. At the lower frequency, the ^1^H-NMR spectrum displayed an oxygenated methine proton δ_H_ 4.44 (1H, t, *J* = 8.8 Hz, H-8′′), four methylene protons [δ_H_ 2.98 (1H, dd, *J* = 15.2, 8.8 Hz, H-7′′a), 2.92 (1H, dd, *J* = 15.2, 10.0 Hz, H-7′′b), 3.12 (1H, d, *J* = 13.5 Hz, H-5a) and 3.25 (1H, d, *J* = 13.5 Hz, H-5b)] and two methyl signals δ_H_ 1.06 (3H, s, H-10′′) and 1.07 (3H, s, H-11′′). The ^13^C-NMR spectrum showed 22 carbon signals corresponding to a carbonyl carbon, fourteen signals in the sp^2^ region of δ_C_ 116.1–164.3, three oxygenated carbons including a hemiacetal carbon δ_C_ 108.0 (C-4), two methylene groups and two methyl groups. Comparison of the NMR data of compound **1** with those of butyrolactone IV (**7**) suggested that they shared the same core frame unit except that the substitution of C-4 in **1** was a hydroxyl group and C-2 was unsubstituted. The structure was further determined by HMBC spectrum (Figure 2), the correlations from H-2 to C-1, C-3, C-4 and C-1′, from H_2_-5 to C-3, C-4 and C-6′′, as well as from H_2_-7′′ to C-3′′, C-4′′ and C-8′′ confirmed the assignment of the α,β-unsaturated-γ-lactone moiety. On the basis of these data, the planar structure of compound **1** was established.

The absolute configuration of the C-4 tertiary alcohol was deduced by the circular dichroism (CD) data of the in situ formed [Rh_2_(OCOCF_3_)_4_] complex, with the inherent contribution subtracted. A positive Cotton effect at 350 nm in the Rh_2_(OCOCF_3_)_4_-induced CD spectrum indicated the 4*S* absolute configuration on the basis of the bulkiness rule of **1** [18,19]. 

Compound **2** was obtained as a light brownish gum. Its positive HR-ESI-MS showed an ion peak at *m*/*z* 405.1283 [M + Na]^+^ (calcd. 405.1314), indicating the same molecular formula of C_22_H_22_O_6_ as **1**. ^1^H- and ^13^C-NMR spectra of **2** were very similar to those of **1** except the signals of the lactone moiety. Combined with the HSQC spectrum, the ^1^H-NMR data of δ_H_ 6.54 (d, *J* = 8.6 Hz, H-4) in **2** was assigned to be a hemiacetal proton rather than an olefinic one. The ^13^C-NMR data of C-2 had shifted downfield (δ_C_ 124.1) and C-3 and C-4 shifted upfield (δ_C_ 156.4, 97.8), respectively, compared with those of **1** (δ_C_ 112.9, 164.3 and 108.0). The above information illustrated the α,β-unsaturated-γ-lactone was isomerized, which was confirmed by the HMBC correlations from H-4 to C-1 and C-2, from 4-OH to C-4 and C-3, as well as from H_2_-5 to C-2, C-3 and C-6′′ as shown in Figure 2.

The determination of the absolute configuration of C-4 was based on the CD analysis. The CD of the α,β*-*unsaturated γ-lactone rings with a chiral γ-carbon shows Cotton effects associated with the π→π* transition in the region 200–235 nm [20] and the n→π* transition in the region 235–270 nm [20,21,22,23]. Compound **2** showed positive π→π* Cotton effect at 223 nm and negative n→π* Cotton effect at 275 nm, indicating the *S* absolute configuration at C-4 in **2 [20,21]**.

**Figure 3:**
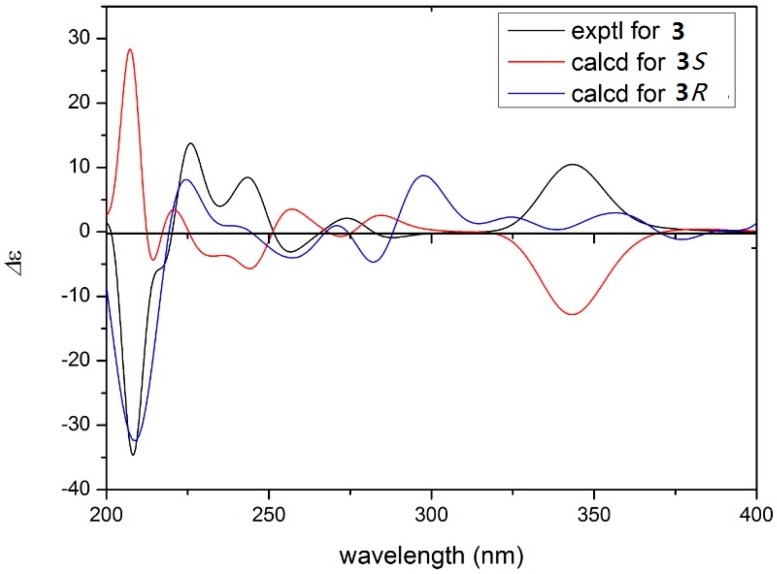
Calculated and experimental circular dichroism (CD) spectra of compound **3**.

Compound **3** was also obtained as a light brownish gum. It has the molecular formula C_22_H_22_O_5_, as deduced from the HR-ESI-MS data [M + Na]^+^ at *m*/*z* 389.1367 (calcd. 389.1365). The formula differs from that of **2** by the loss of one oxygen atom, suggesting the absence of a hydroxyl group for **3**. The NMR data of **3** were very similar to those of **2** and the difference was the side chain moiety was an isopentenyl instead of a furan ring. The ^1^H-NMR spectrum of **3** indicated the presence of two methylene protons at δ_H_ 3.14 (2H, d, *J* = 7.2 Hz, H_2_-7′′), an olefinic proton at δ_H_ 5.21 (1H, t, *J* = 7.2 Hz, H-8′′) and two methyl signals at δ_H_ 1.62 (3H, s, H-10′′) and 1.66 (3H, s, H-11′′), which suggested the existence of an isopentenyl group. The planar construction of **3** was determined by the HMBC correlations of H-4/C-1, C-2; H_2_-5/C-2, C-3, C-6′′; H-7′′/C-8′′, C-2′′, C-3′′ (Figure 2). The absolute configuration of **3** was determined as 4*R* by comparison of its experimental electronic CD curve with that calculated (Figure 3).

The antioxidant activities of **1**−**10** were evaluated by their ability to scavenge DPPH and ABTS radicals (Table 2). Compounds **4**–**10** showed significant DPPH radical scavenging activities with IC_50_ values of 15.9 to 34.3 μM, whereas compounds **1**–**3** (IC_50_ > 100 μM) did not display any DPPH radical scavenging activity (Table 2). In the ABTS assay, most of the isolated compounds exhibited significant antioxidant activity, with compounds **2** and **4**−**10** being stronger than that of the standard compound ascorbic acid (IC_50_ = 12.5 μM), whereas compounds **1** and **3** exhibited moderate ABTS radical-scavenging activities (IC_50_ = 33.1 and 29.5 μM). A comparison of the structures of **1**–**3** with those of **4**–**10** indicated that an enolic hydroxyl situated at the α,β-unsaturated-γ-lactone ring and the phenolic hydroxyl were crucial for the antioxidant activity, which was consistent with previous observations [24,25].

The isolated compounds were tested for their in vitro cytotoxicities using four tumor cell lines: HL-60, ASPC1, HCT-116 and PC-3. The resulting IC_50_ values are listed in Table 3. 5-Fluorouracil was used as a positive control and its IC_50_ values were 6.38, 2.70, 7.77 and 15.6 μM, respectively. Compounds **2**, **4**, **7** and **10** exhibited potent antitumor activity against HL-60 cell line with IC_50_ values ranging from 13.2 to 41.6 μM. Compounds **4** and **8** showed moderate cytotoxity against PC-3 cell line with IC_50_ values of 41.7 and 58.3 μM, respectively. Only compound **8** displayed weak cytotoxic activity against HCT-116 cell line with an IC_50_ of 41.7 μM and all tested isolates were inactive against the ASPC1 cell line. The antimicrobial activity of compounds **1**–**10** were assessed towards *Bacillus subtilis*, *Staphylococcus aureus*, *Escherichia coli* and *Candida albicans*. However, only compound **10** presented significant antimicrobial activity against *Staphylococcus aureus* with minimum inhibitory concentration (MIC) of 21.3 μM, and chloramphenicol was used as positive control with the MIC of 12.1 μM.

## 3. Experimental Section

### 3.1. General Experimental Procedures

Optical rotations were determined on a Anton Paar MCP200 automatic polarimeter (Anton Paar GmbH, Graz, Austria). UV spectra were recorded on a Shimadzu-2201 (Kyoto, Japan). The HR-ESI-MS data were obtained on a microTOF-Q Bruker mass instrument (Bruker Daltonics, Billerica, MA, USA). HR-ESI-MS data were measured on a Micro-mass Autospec-UntimaE TOF mass spectrophotometer (Waters, Milford, MA, USA). CD spectra were recorded with a BioLogic MOS-450 spectrometer (BioLogic Science, Grenoble, France). NMR spectra were run on a Bruker AVANCE-400/-600 spectrometer (Bruker BioSpin GmbH, Rheinstetten, Germany). Column chromatography was performed on Silic gel G (200–300 mesh; Qingdao Haiyang Chemical Factory, Qingdao, China) and Sephadex LH-20 (Pharmacia, Piscataway, NJ, USA) columns. Thin layer chromatography was carried out using Silic gel GF254 (Qingdao Haiyang Chemical Factory) plates. HPLC was performed using a Shimadzu LC-10AVP liquid chromatography using YMC-pack C_18_ (ODS) column (10 × 250 mm, 5 μm, Tokyo, Japan).

### 3.2. Microorganism

The fungus CBS-P-2 was obtained from the volcanic soil sample of Changbai Mountain in Jilin province, China. The fungus was identified as *Aspergillus* sp. on the basis of morphological and molecular taxonomic methods and was deposited at the School of Traditional Chinese Materia Medica, Shenyang Pharmaceutical University, China.

### 3.3. Extraction and Isolation

The fresh mycelia of *Aspergillus* sp. CBS-P-2 grew on potato dextrose agar medium at 28 °C for three days. Agar plugs were inoculated in a 500 mL Erlenmeyer flask containing 200 mL of media (20% potato, 2% maltose, 2% mannitol, 1% d-glucose, 0.5% monosodium glutamate, 0.5% peptone, 0.3% yeast extract) before sterilization, and incubated statically at 25 °C. After 30 days, the fermented broth (80 L) was filtered through cheesecloth to be separated into the supernatant and the mycelia.

The fermentation broth was concentrated and extracted three times with ethyl acetate. The ethyl acetate extract (70 g) was fractionated by silica gel column chromatography eluting with CH_2_Cl_2_–MeOH (100:0–0:100, *v/v*) to obtain nine fractions (Fr. 1–9) based on TLC analyses. Fr. 7 (10 g) was subjected to CC on silica gel columns using a stepwise solvent gradient method with petroleum ether (PE)/EtOAc (100:0–0:100) to give eight sub-fractions (Fr. 7.1–7.8). Fr. 7.6 (260 mg) was chromatographed over Sephadex LH-20 eluting with MeOH, followed by semi-preparative HPLC (56% CH_3_OH/H_2_O, a flow rate at 3 mL/min, 210 nm) to yield compounds **3** (8.9 mg, t_R_ 61 min), **9** (34.6 mg, t_R_ 40 min), **10** (4.5 mg, t_R_ 67 min) and **4** (6.3 mg, t_R_ 75 min). Fr. 7.7 (6.5 g) was purified by semi-preparative HPLC (41% CH_3_CN/H_2_O, a flow rate at 3 mL/min, 210 nm) to yield compounds **6** (2 g*,* t_R_ 28 min), **7** (1 g, t_R_ 58 min), **5** (1 g, t_R_ 63 min) and **8** (12 mg, t_R_ 150 min). Fr. 7.8 (400 mg) was subjected to Sephadex LH-20 (4 × 34 cm) eluting with CH_3_OH, then by the following semi-preparative HPLC (35% CH_3_CN/H_2_O and 0.1% CF_3_COOH, a flow rate at 3 mL/min, 210 nm) to yield compounds **1** (5.7 mg, t_R_ 34 min) and **2** (8.8 mg, t_R_ 36 min).

### 3.4. Spectroscopic Data

*Aspernolides H* (**1**): light yellowish gum, [α]${}_{D}^{20}$ = −18.7 (*c* 0.32, MeOH); UV (MeOH) λ_max_ (log ε): 202 (4.42) nm (aromatic group); IR (KBr) ν_max_ cm^−1^: 3407, 2948, 2843, 1640, 1454, 1384, 1113, 1053; ^1^H-NMR (DMSO-*d*_6_, 400 MHz) and ^13^C-NMR (DMSO-*d*_6_, 100 MHz) data see Table 1; HR-ESI-MS *m*/*z*: 405.1300 [M + Na]^+^ (calcd. for C_22_H_22_NaO_6_, 405.1314). 

*Aspernolides I* (**2**): light brownish gum, [α]${}_{D}^{20}$ = −16.7 (*c* 0.33, MeOH); UV (MeOH) λ_max_ (log ε): 300 (3.96) (carbonyl group), 204 (4.35) nm (aromatic group); IR (KBr) ν_max_ cm^−1^: 3393, 2947, 2834, 1648, 1450, 1385, 1114, 1032; ^1^H-NMR (DMSO-*d*_6_, 400 MHz) and ^13^C-NMR (DMSO-*d*_6_, 100 MHz) data see Table 1; HR-ESI-MS *m*/*z*: 405.1283 [M + Na]^+^ (calcd. for C_22_H_22_NaO_6_, 405.1314).

*Aspernolides J* (**3**): light brownish gum, [α]${}_{D}^{20}$ = +2.57 (*c* 1.36, MeOH); UV (MeOH) λ_max_ (log ε): 216 (4.08) nm (aromatic group); IR (KBr) ν_max_ cm^−1^: 3407, 2948, 1640, 1454, 1385, 1114, 1019; ^1^H-NMR (DMSO-*d*_6_, 400 MHz) and ^13^C-NMR (DMSO-*d*_6_, 100 MHz) data see Table 1; HR-ESI-MS *m*/*z*: 389.1365 [M + Na]^+^ (calcd. for C_24_H_24_NaO_4_, 389.1367).

*3-Hydroxy-5-[[4-hydroxy-3-(3-methyl-2-buten-1-yl)phenyl]methyl]-4-(4-hydroxyphenyl)-2(5H)-furanone* (**10**): light brownish gum, [α]${}_{D}^{20}$ = −28.6 (*c* 0.28, MeOH); ^1^H-NMR (DMSO-*d*_6_, 400 MHz) δ 9.85 (1H, brs, H-4’), 9.15 (1H, brs, H-4’’), 7.54 (2H, d, *J* = 8.8 Hz, H-2’, 6’), 6.88 (2H, d, *J* = 8.8 Hz, H-3’, 5’), 6.63 (1H, dd, *J* = 8.0, 2.0 Hz, H-6’’), 6.59 (1H, d, *J* = 8.0 Hz, H-5’’), 6.55 (1H, brs, H-2’’), 5.63 (1H, dd, *J* = 5.6, 3.6 Hz, H-4), 5.12 (1H, t, *J* = 7.2 Hz, H-8’’), 3.14 (1H, dd, *J* = 14.8, 5.6 Hz, H-5a), 2.71 (1H, dd, *J* = 14.8, 3.6 Hz, H-5b), 1.65 (3H, s, H-10’’), 1.60 (3H, s, H-11’’); ^13^C-NMR (DMSO-*d*_6_, 100 MHz) δ 169.6 (C-1), 158.2 (C-4’), 153.9 (C-4’’), 136.7 (C-2), 131.4 (C-2’’), 130.9 (C-8’’), 130.1 (C-3), 129.4 (C-2’’), 128.3 (C-6’’), 127.1 (C-9’’), 125.6 (C-1’’), 123.2 (C-1’’), 122.2 (C-1’), 116.0 (C-3’, 5’), 114.7 (C-5’’), 78.3 (C-4), 38.6 (C-5), 28.3 (C-7’’), 25.9 (C-11’’), 18.1 (C-10’’).

The ^1^H- and ^13^C-NMR data of **1**–**10**, HR-ESI-MS, 2D-NMR spectra of compounds **1**–**3** are available as Supplementary Materials.

### 3.5. Biological Activity

#### 3.5.1. Cytotoxicity Assay

Cytotoxic activities of the isolates were evaluated by the trypan blue method [26,27] against the human leukaemia cell lines (HL-60), and the MTT assay [28] against the human colon cell lines (HCT-116), pancreatic cancer cell lines (ASPC1) and prostate cancer cell lines (PC-3). The cell lines were purchased from America Type Culture Collection, ATCC (Rockville, MD, USA) and cultured in Roswell Park Memorial Institute (RPMI)-1640 medium (Gibco, New York, NY, USA) supplemented with 100 U/mL penicillin, 100 µg/mL streptomycin, 1 mM glutamine and 10% heat-inactivated foetal bovine serum (Gibco) at 37 °C in humidified atmosphere with 5% CO_2_. Appropriate dilutions of the test metabolites were added to the cultures. The growth inhibition was calculated comparing with control cells (5-Fluorouracil was used as positive control) and a half growth inhibitory concentration (IC_50_) was obtained by regression analysis of the concentration response data. The cytotoxicity of 5-Fluorouracil against the HL-60, ASPC1, PC-3 and HCT-116 cell lines were estimated by their IC_50_ values of 6.38, 2.70, 7.77 and 15.6 μM, respectively.

#### 3.5.2 Antioxidant Activity

##### 1-Diphenyl-2-picrylhydrazyl Free Radical (DPPH·) Scavenging Assay 

The DPPH· scavenging activity was assessed according to the method described with minor modifications with lower DPPH· concentration (0.2 mM) and different sample to DPPH ratio (1:1) [29]. To a 100 µL aliquot of the sample with different concentrations was added 100 µL of DPPH solution (0.2 mM) in a 96-well microplate. The mixture was shaken vigorously and incubated in darkness for 30 min. The absorbance of the reaction solution at 517 nm was recorded using a Varioskan flash multimode reader (Thermo, Waltham, MA, USA). Ascorbic acid was used as a positive control. The percentage of scavenging DPPH versus concentration was plotted using the following equation: DPPH· scavenging activity (%) = [1 − (S − SB)/(C − CB)] × 100%, where S, SB, C and CB are the absorption of the sample, the blank sample, the control and the blank control, respectively.

##### 2,2′-Azinobis(3-ethylbenzothiazoline-6-sulfonate) Free Radical (ABTS^•+^) Scavenging

The total antioxidant capacity was evaluated using an improved ABTS radical cation (ABTS^•+^) decolorization assay with some modifications [30]. ABTS^•+^ was generated by reacting 7 mM aqueous solution of ABTS^•+^ with 2.45 mM K_2_S_2_O_8_ and putting the mixture in the dark at room temperature for 16 h before use. The ABTS^•+^ solution was diluted with ethanol, to an absorbance of 0.7 ± 0.02 at 734 nm. An ethanolic solution (100 μL) of different concentrations of samples (0.1−50 μM) was mixed with 150 μL of diluted ABTS^•+^ solution. After reacting at room temperature for 10 min, the absorbance was measured at 734 nm using a molecular devices versa max microplate reader (Thermo Scientific, Waltham, MA, USA). The ABTS^•+^ scavenging ability was calculated using the formula ABTS^•+^ scavenging activity (%) = [1 − (S − SB)/(C − CB)] × 100%, where S, S B, C, and CB are the absorption of the sample, the blank sample, the control, and the blank control, respectively. Tests were performed in triplicate.

#### 3.5.3. Antimicrobial Bioassay

The MIC values for all metabolites were determined by the dilution method. For sample preparation, each of the test compounds was dissolved in DMSO and then diluted with sterile broth to a concentration of 500 μg/mL. Further dilutions of the compounds in the test medium were prepared at the required quantities of 250, 125, etc. down to 3.9 μg/mL. Chloramphenicol and fluconazole were used as positive controls for bacteria and fungus, respectively. The in vitro antimicrobial activity of the compounds was tested by tube-dilution technique using individually packaged, flat bottomed, 96-well microtitre plates (Corning Incorporated, Corning, NY, USA) (Clinical and Laboratory Standards Institute, NCCLS, 2000). Bacterial strains were maintained on luria bertani (LB) medium for 48 h at 37 °C and fungal strains were on Potato Destrose Agar (PDA) medium for 48 h at 28 °C. The cell density for bacteria was 2–4 × 10^7^ CFU/mL and 2–4 × 10^5^ CFU/mL for fungus. A serial dilution of compounds were performed in the microplates and incubated for 12 h. The last tube with no growth of microorganism was recorded to represent the MIC value expressed in μg/mL. 

## 4. Conclusions

Chemical investigations of *Aspergillus* sp. CBS-P-2 afforded three new butenolides aspernolides H-J (**1**–**3**) and seven known ones **4**–**10**. Chemical structures of the isolated compounds were identified on the basis of 1D-, 2D-NMR, HR-ESI-MS, CD, and IR spectroscopic data. All the compounds were evaluated for the antioxidant activity, the cytotoxity against four human tumor cell lines and antimicrobial activities. Most isolates showed significant capacity for scavenging DPPH and ABTS free radicals, which proved butenolides to be potent antioxidant agents. Furthermore, compounds **2**, **4**, **7** and **10** showed potent cytotoxic activities against HL-60 cell lines. Compound **10** showed strong antimicrobial activity against *Staphylococcus aureus* with MIC of 21.3 μM. These results provide further understanding about the chemistry and bioactivities of the butenolide derivatives.

## Figures and Tables

**Table 1:** ^1^H-NMR (400 MHz) and ^13^C-NMR (100 MHz) spectral data of compounds **1**, **2** and **3** in DMSO-*d*_6_.

Position	1		2		3	
	δ_H_ (*J* in Hz)	δ_C_	δ_H_ (*J* in Hz)	δ_C_	δ_H_ (*J* in Hz)	δ_C_
1		170.3		172.9		172.4
2	6.23 (s)	112.9		124.1		124.3
3		164.3		156.4		156.6
4		108	6.54 (d, 8.6)	97.8	6.52 (d, 8.4)	97.7
5	3.12 (d, 13.5)	43.8	3.65 (d, 15.4)	29.2	3.57 (d, 15.6)	29
	3.25 (d, 13.5)		3.74 (d, 15.4)		3.68 (d, 15.6)	
1′		121.2		122		122.1
2′	7.74 (d, 8.4)	130.8	7.45 (d, 8.8)	130.9	7.44 (d, 8.8)	130.8
3′	6.89 (d, 8.4)	116.3	6.84 (d, 8.8)	116.2	6.82 (d, 8.8)	116
4′		160.6		158.9		159.8
5′	6.89 (d, 8.4)	116.3	6.84 (d, 8.8)	116.2	6.82 (d, 8.8)	116
6′	7.74 (d, 8.4)	130.8	7.45 (d, 8.8)	130.9	7.44 (d, 8.8)	130.8
1′′		126.1		129.4		128.1
2′′	6.48 (brs)	129.6	7.02 (brs)	124.8	6.88 (d, 2.0)	129.5
3′′		127.3		128.3		128.8
4′′		159.1		159		154.1
5′′	6.46 (d, 8.4)	116.1	6.64 (d, 8.4)	109	6.67 (d, 8.0)	115.3
6′′	6.54 (brd, 8.4)	129.7	6.89 (brd, 8.4)	127.4	6.80 (dd, 8.8, 2.0)	126.3
7′′	2.98 (dd, 15.2, 8.8)	30.2	3.07 (2H, m)	30.4	3.14 (2H, d, 7.2)	28.4
	2.92 (dd, 15.2, 10.0)					
8′′	4.44 (t, 8.8)	89.4	4.49 (t, 9.0)	89.5	5.21 (t, 7.2)	123.2
9′′		70.4		70		131.7
10′′	1.06 (3H,s)	25.3	1.12 (3H, s)	25.4	1.62 (3H, s)	18
11′′	1.07 (3H,s)	26.5	1.12 (3H, s)	26.6	1.66 (3H, s)	25.9
4-OH	7.91 (brs)		7.76 (d, 8.8)		7.77 (d, 8.4)	
4′-OH	10.26 (brs)		10.07 (brs)		10.06 (brs)	
4′′-OH					9.15 (s)	

**Table 2:** Antioxidant activities of compounds **1**–**10** (IC_50_, μM).

Compounds	Antioxidant Activities	
	DPPH	ABTS
**1**	>100	33.1 ± 1.12
**2**	>100	9.1 ± 1.21
**3**	>100	29.5 ± 1.11
**4**	34.1 ± 0.3	2.8 ± 0.11
**5**	25.6 ± 0.36	5.0 ± 0.7
**6**	30.0 ± 1.25	6.6 ± 0.6
**7**	15.9 ± 0.56	5.1 ± 0.3
**8**	25.6 ± 1.8	6.2 ± 0.3
**9**	20.7 ± 3.65	3.7 ± 0.08
**10**	34.3 ± 0.03	5.3 ± 0.05
Ascorbic acid ^a^	25.1 ± 0.18	12.5 ± 0.02

^a^ Ascorbic acid was used as positive control in test of antioxidant activities.

**Table 3:** Cytotoxicity of compounds **1**–**10** against four tumor cell lines.

Compounds	IC_50_ (μM)			
	HL-60	ASPC1	PC-3	HCT-116
**1**	>80	>80	>80	>80
**2**	39.4	>80	>80	>80
**3**	>80	>80	>80	>80
**4**	13.2	>80	41.7	>80
**5**	>80	>80	>80	>80
**6**	>80	>80	>80	>80
**7**	41.6	>80	>80	>80
**8**	>80	>80	58.3	66.9
**9**	>80	>80	>80	>80
**10**	16.3	>80	>80	>80
5-Fluorouracil ^a^	6.38	2.70	7.77	15.6

^a^ 5-Fluorouracil was used as positive control. Data expressed as IC_50_ values (μM). HL-60, human leukaemia cell; ASPC1, pancreatic cancer cell; HCT-116, human colon cell lines; PC-3, human prostate cancer cell.

## Supplementary Materials

Supplementary materials can be accessed at: http://www.mdpi.com/1420-3049/21/10/1361/s1.
