# Supplementary material for: Three New Butenolides from the Fungus Aspergillus sp. CBS-P-2
Source: Molecules. 2016 Oct 13;21(10):1361. doi: 10.3390/molecules21101361 (PMC6273075; doi:10.3390/molecules21101361)
Supplement: Supplementary file 1 [file molecules-21-01361-s001.pdf]

Supplementary Materials: Three New Butenolides from the Fungus *Aspergillus* sp. CBS-P-2

Xiao An, Yuehu Pei, Shaofei Chen, Shengge Li, Xiaolong Hu, Gang Chen, Bin Lin and Haifeng Wang

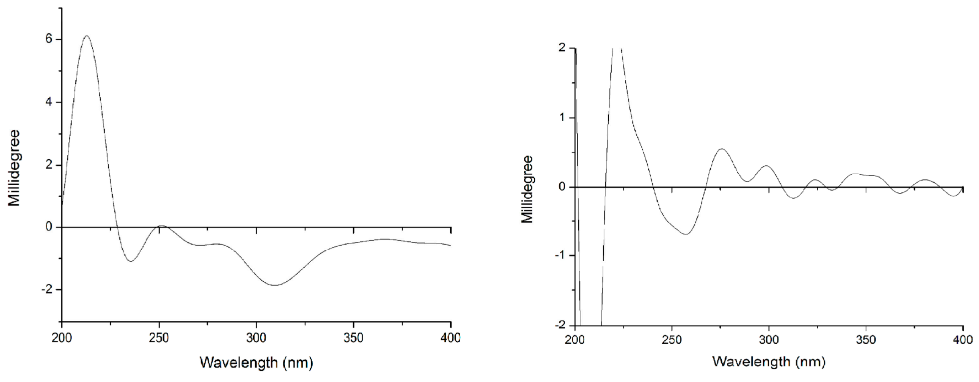

a b

Figure S1. The CD spectrum of **1** and its CD spectrum in $CDCl_3$ of $[Rh_2(OCOCF_3)_4]$ with which the inherent contribution of **1** was subtracted.

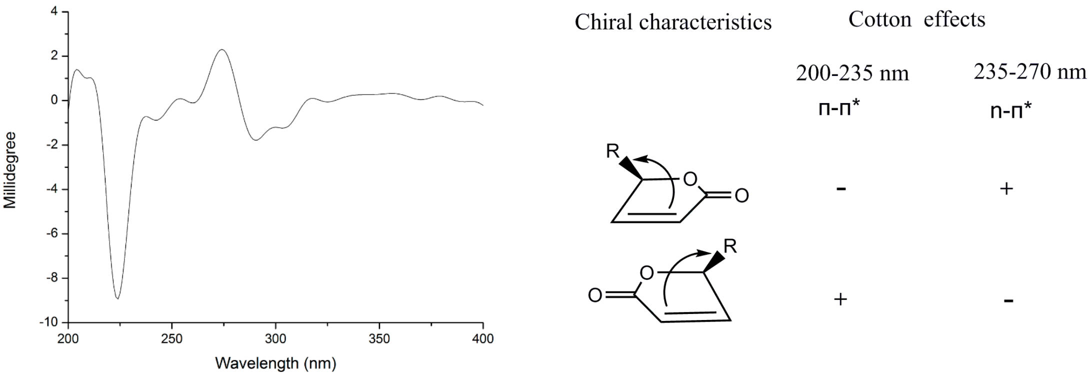

Figure S2. The CD spectrum of **2**.

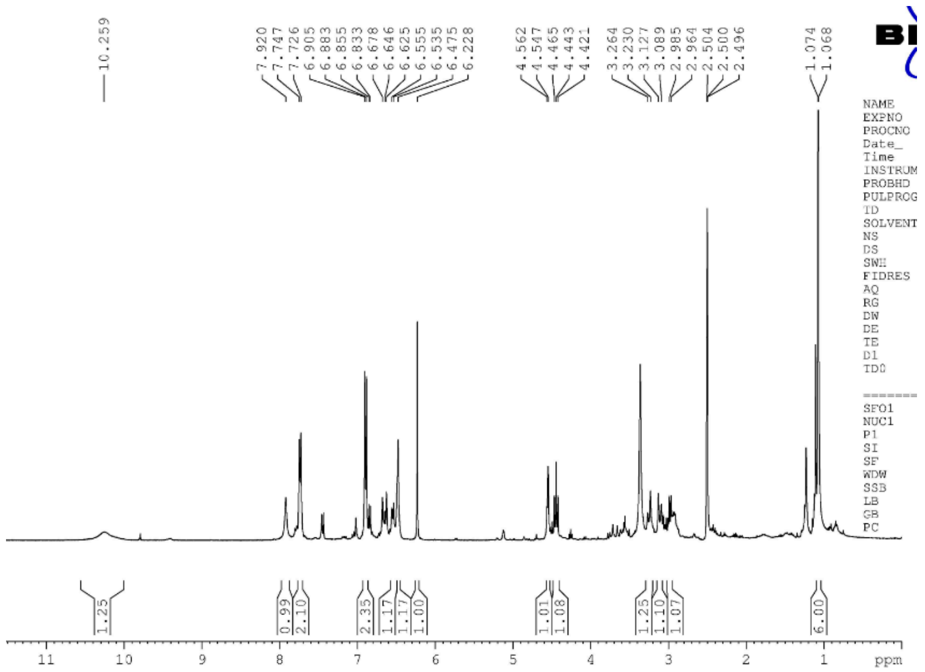

Figure S3. The ^{1}H-NMR (DMSO-d_6, 400 MHz) data of **1**.

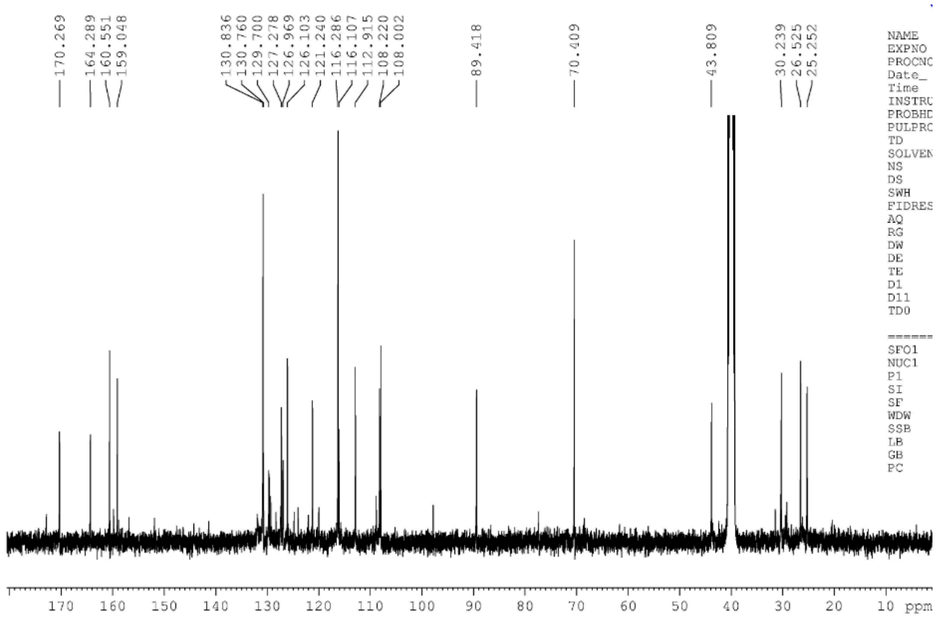

Figure S4. The ^{13}C-NMR (DMSO-d_6, 100 MHz) data of **1**.

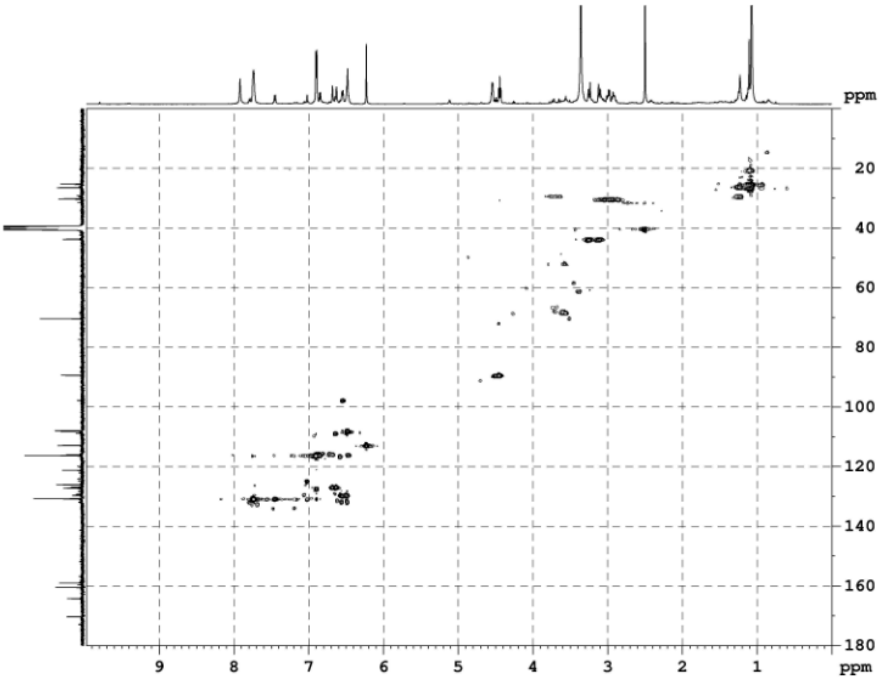

Figure S5. The HSQC spectrum of **1**.

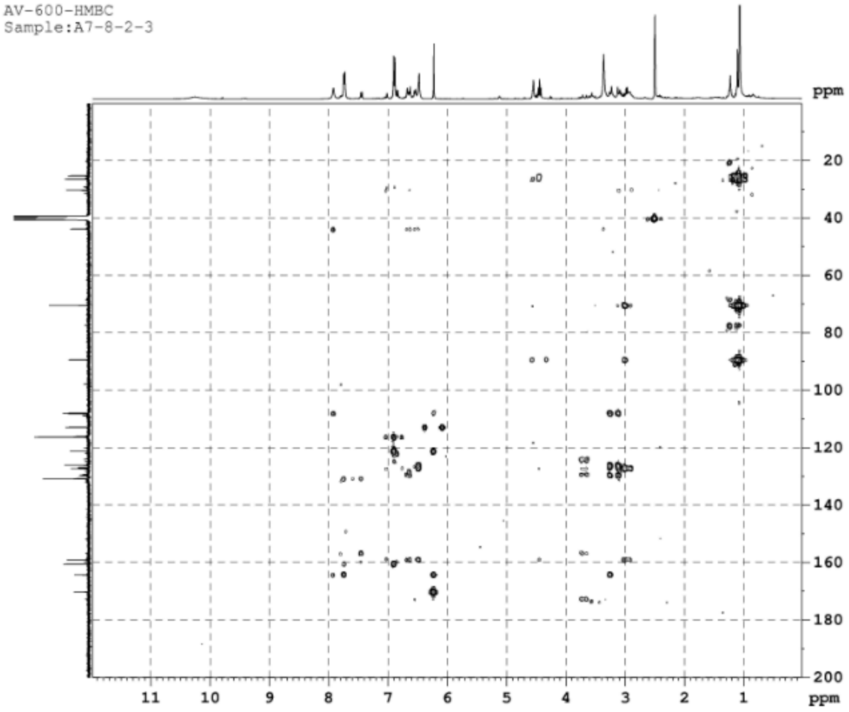

Figure S6. The HMBC spectrum of **1**.

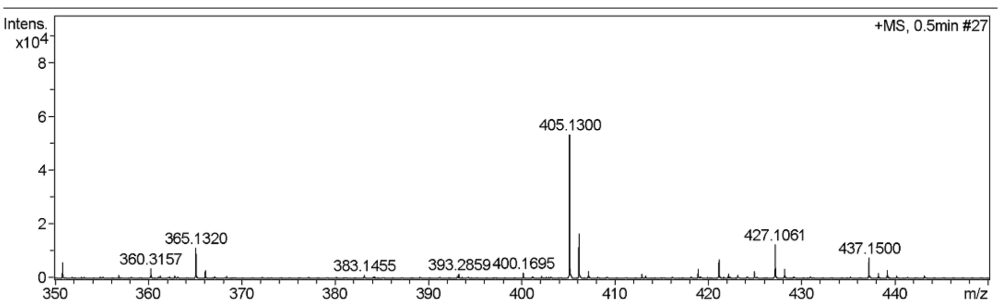

Sum Formula	Sigma	m/z	Err [ppm]	Mean Err [ppm]	Err [mDa]	rdb	N Rule	e⁻
C 22 H 22 Na 1 O 6	0.038	405.1309	2.07	5.39	0.84	11.50	ok	even

Figure S7. The HR-ESI-MS spectrum of **1**.

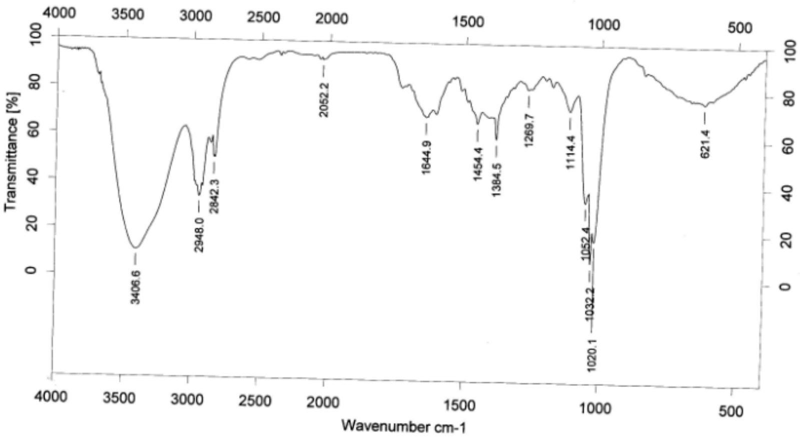

Figure S8. IR spectrum of **1**.

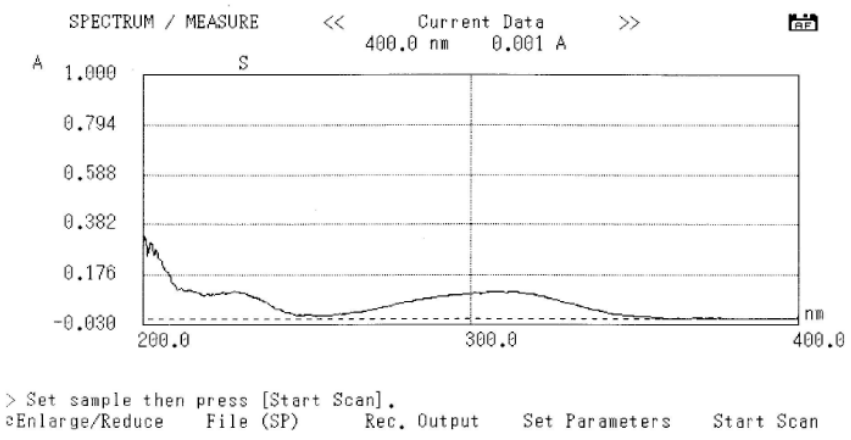

NO.	ABSCISSA	PEAK	HEIGHT	ABSCISSA	VALLEY	HEIGHT
1	227.8	0.1095	0.0209	218.2	0.0906	-0.0200
2	202.4	0.3053	0.0660	212.6	0.1088	-0.0213
3				201.2	0.2547	-0.0578

Figure S9. The UV spectrum of **1**.

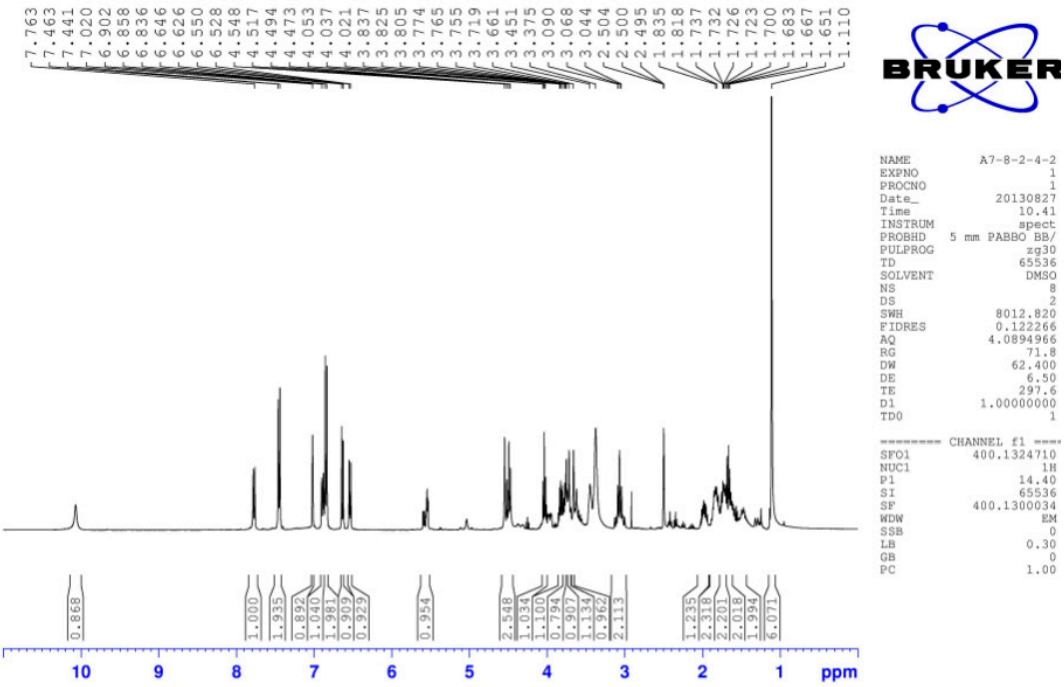

Figure S10. The ^{1}H-NMR (DMSO-d_6, 400 MHz) data of **2**.

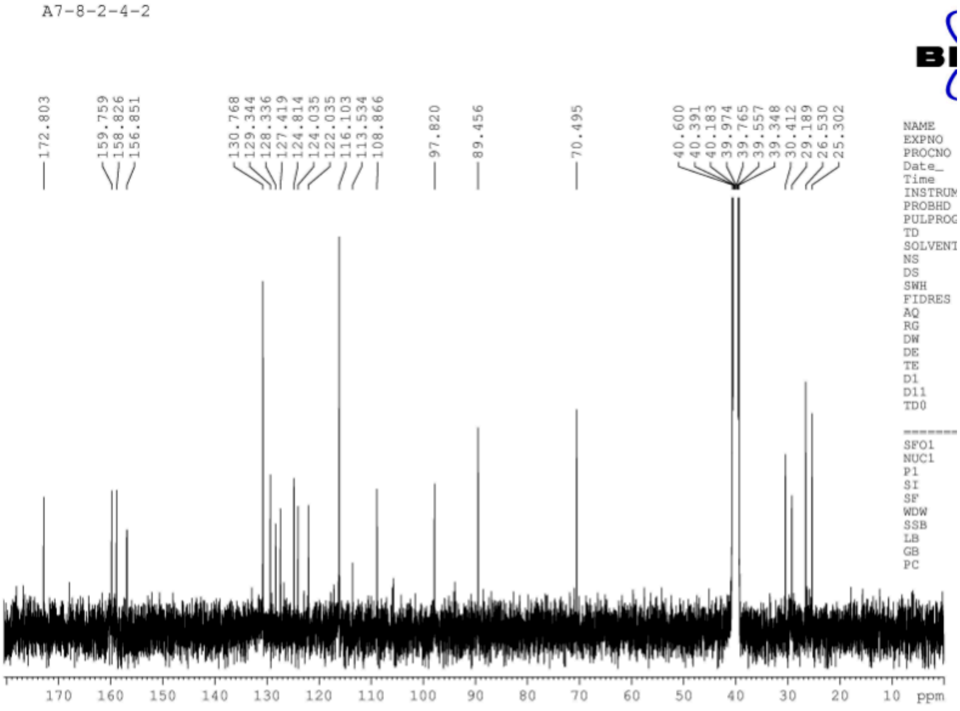

Figure S11. The ^{13}C-NMR (DMSO-d_6, 100 MHz) data of **2**.

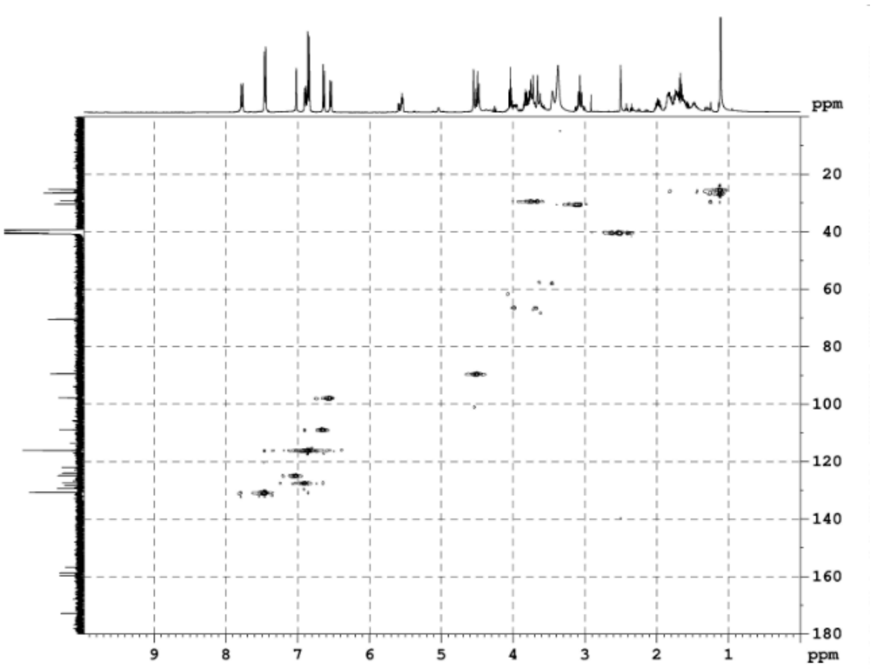

Figure S12. The HSQC spectrum of **2**.

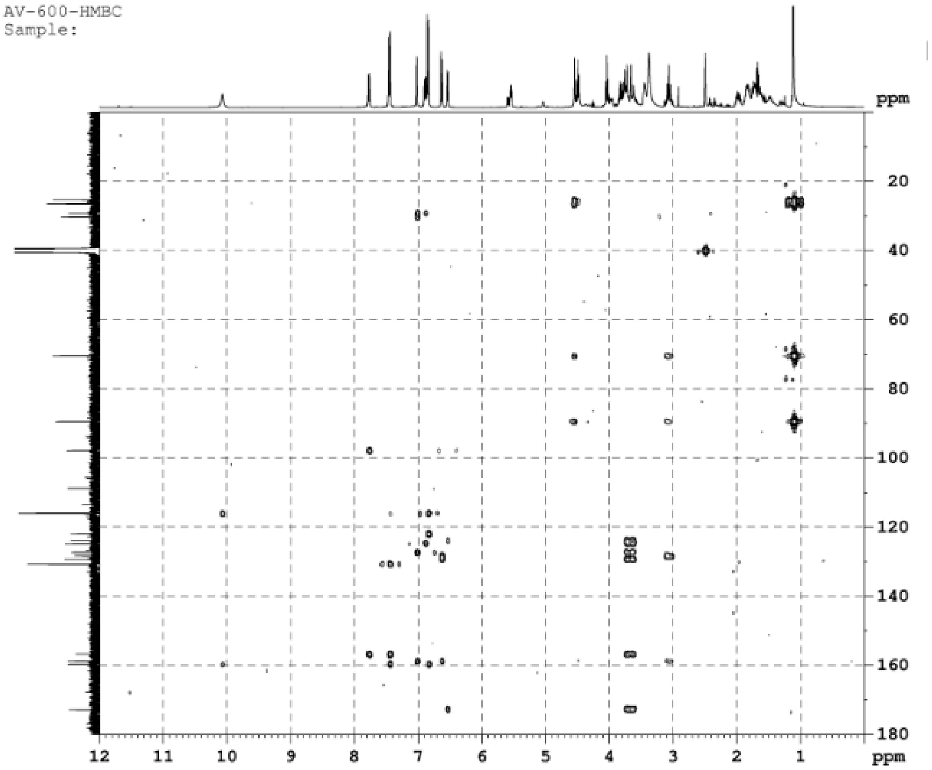

Figure S13. The HMBC spectrum of **2**.

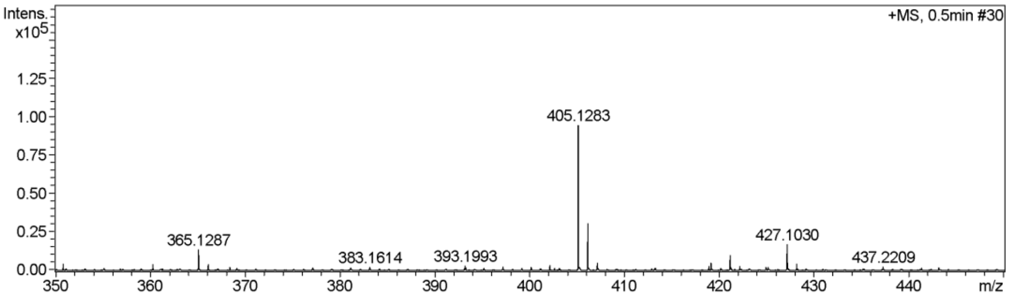

Sum Formula	Sigma	m/z	Err [ppm]	Mean Err [ppm]	Err [mDa]	rdb	N Rule	e⁻
C 22 H 22 Na 1 O 6	0.046	405.1309	6.34	10.30	2.57	11.50	ok	even

Figure S14. The HR-ESI-MS spectrum of **2**.

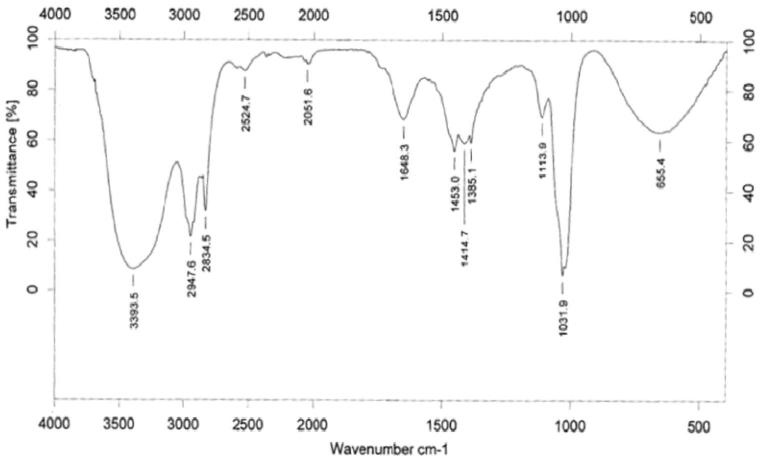

Figure S15. The IR spectrum of **2**.

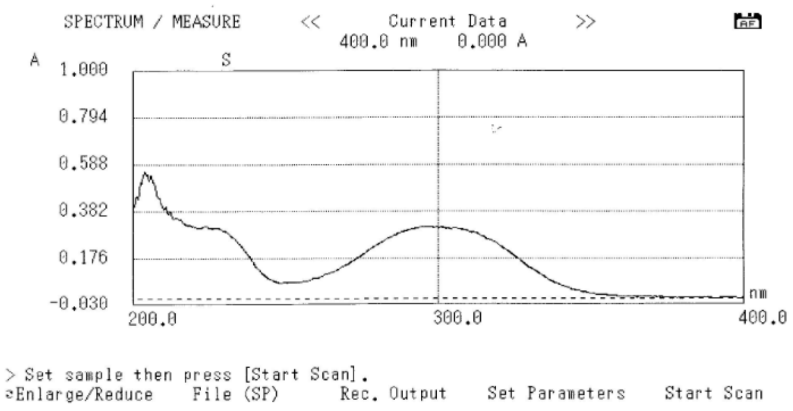

NO.	ABSCISSA	PEAK	HEIGHT	ABSCISSA	VALLEY	HEIGHT
1	226.8	0.3113	0.0136	248.0	0.0679	-0.0551
2	223.6	0.3205	0.0101	211.4	0.3684	-0.0302
3	212.2	0.3825	0.0205			
4	203.6	0.5558	0.1620			

Figure S16. The UV spectrum of **2**.

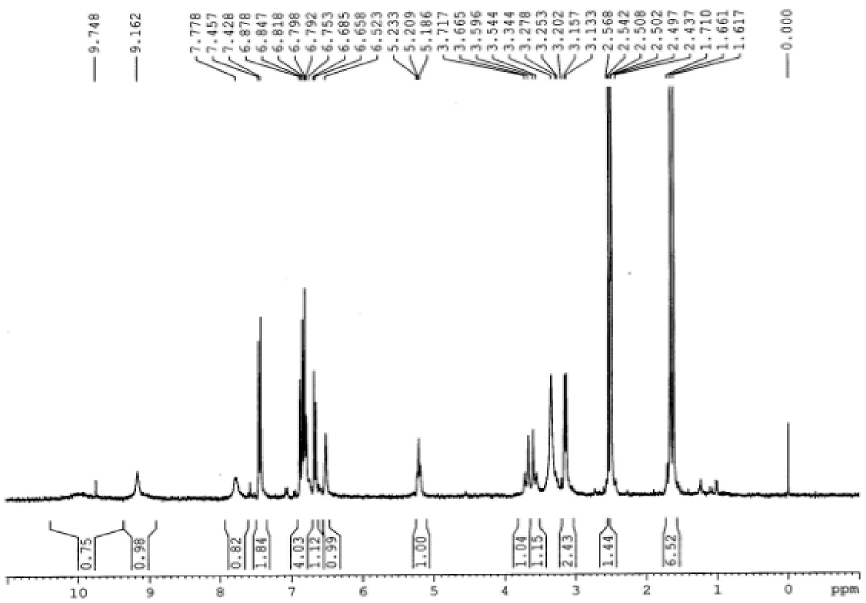

Figure S17. The ^{1}H-NMR (DMSO-d_6, 400 MHz) data of **3**.

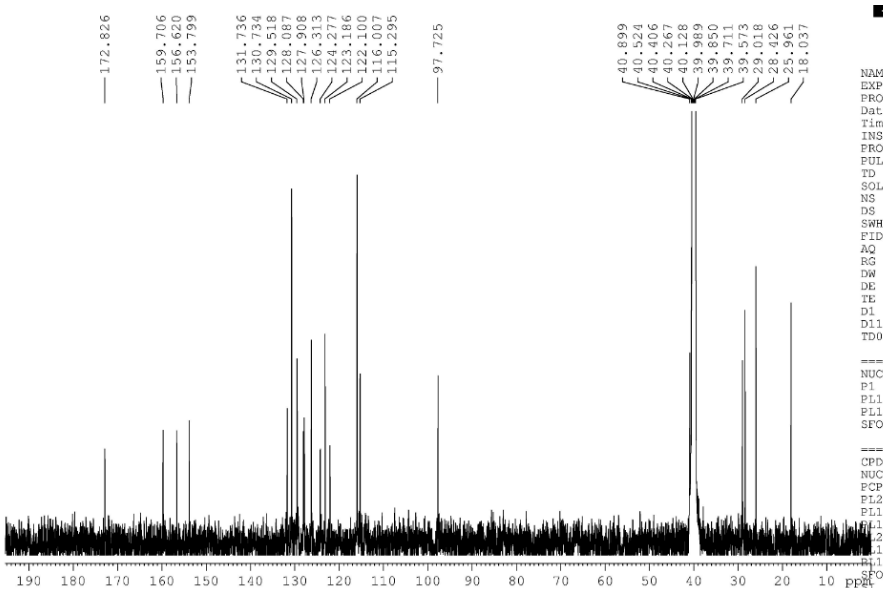

Figure S18. The ^{13}C-NMR (DMSO-d_6, 100 MHz) data of **3**.

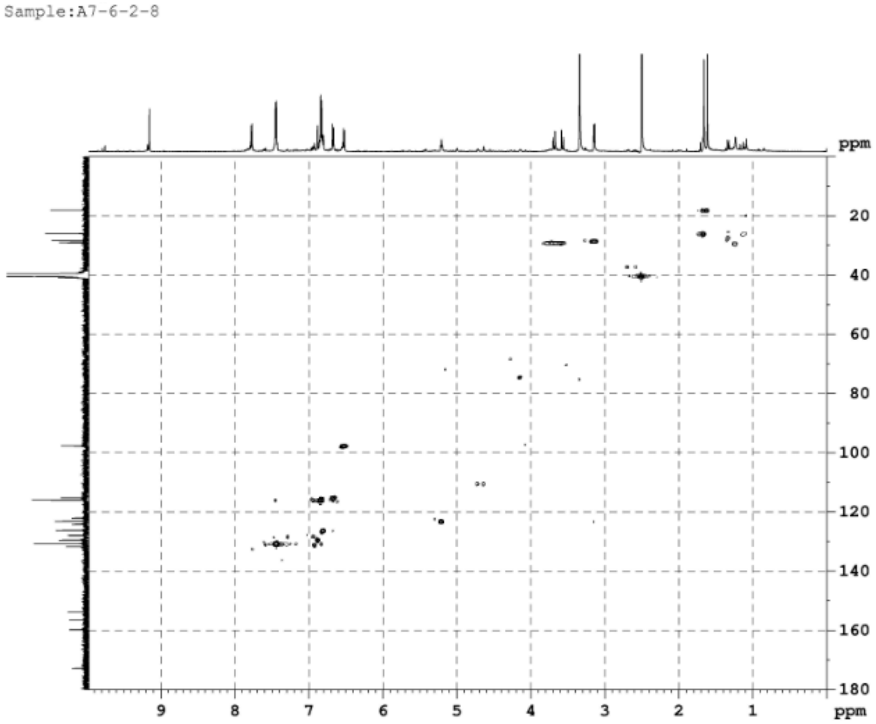

Figure S19. The HSQC spectrum of **3**.

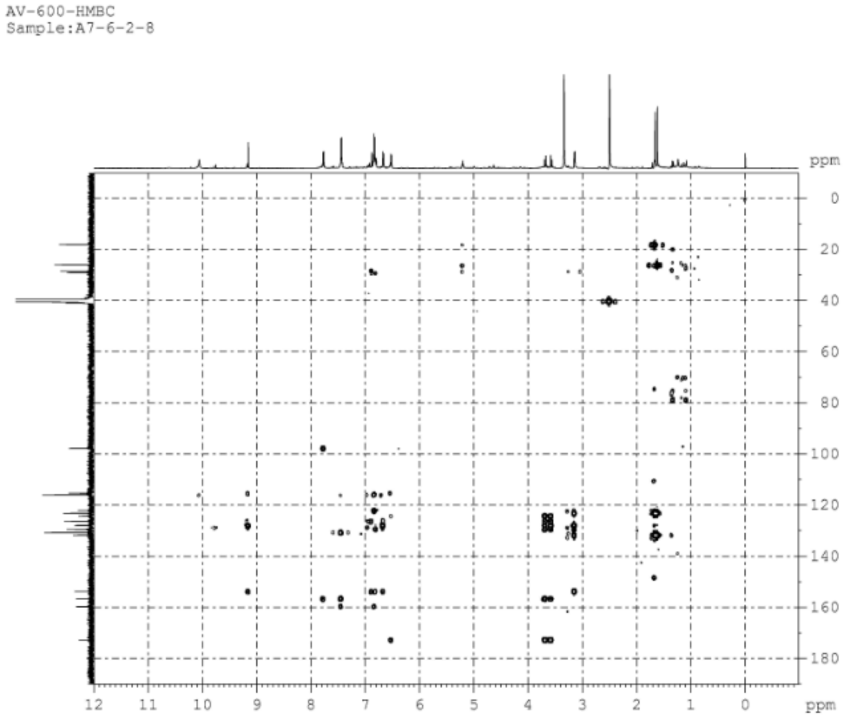

Figure S20. The HMBC spectrum of **3**.

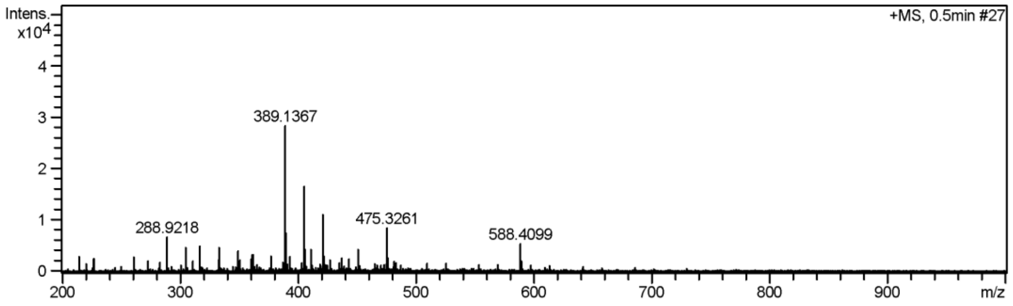

Figure S21. The HR-ESI-MS spectrum of **3**.

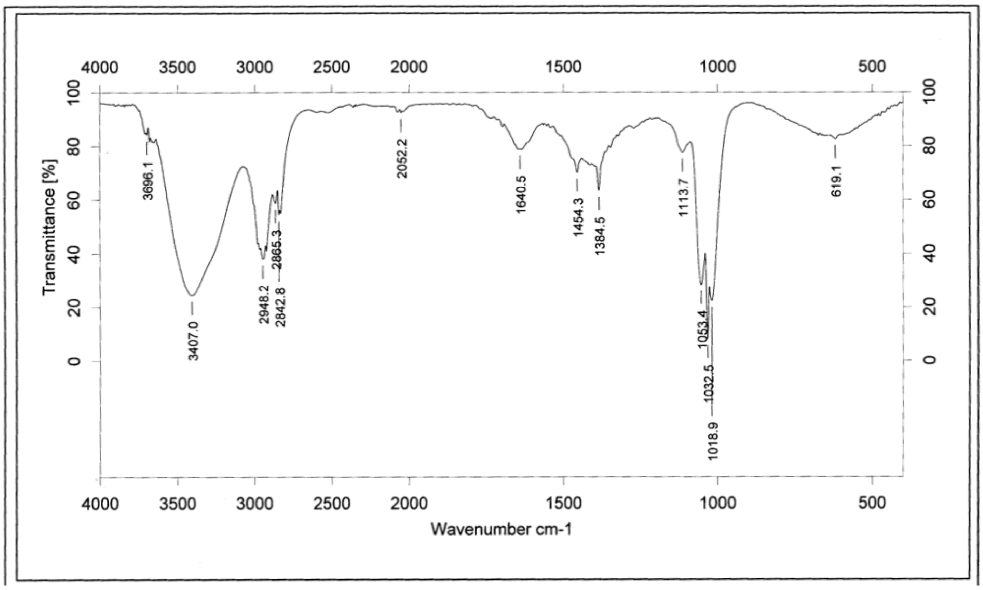

Figure 22. The IR spectrum of **3**.

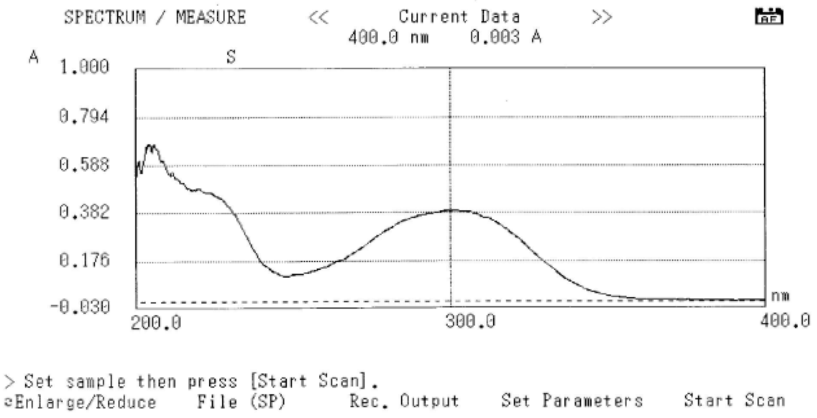

NO.	ABSCISSA	PEAK	HEIGHT	ABSCISSA	VALLEY	HEIGHT
1	301.2	0.3926	0.0141	248.2	0.1097	-0.3428
2	220.2	0.4888	0.0428	217.6	0.4773	-0.0390
3	205.8	0.6805	0.0481	205.0	0.6429	-0.0363
4	204.2	0.6819	0.0579			

Figure S23. The UV spectrum of **3**.

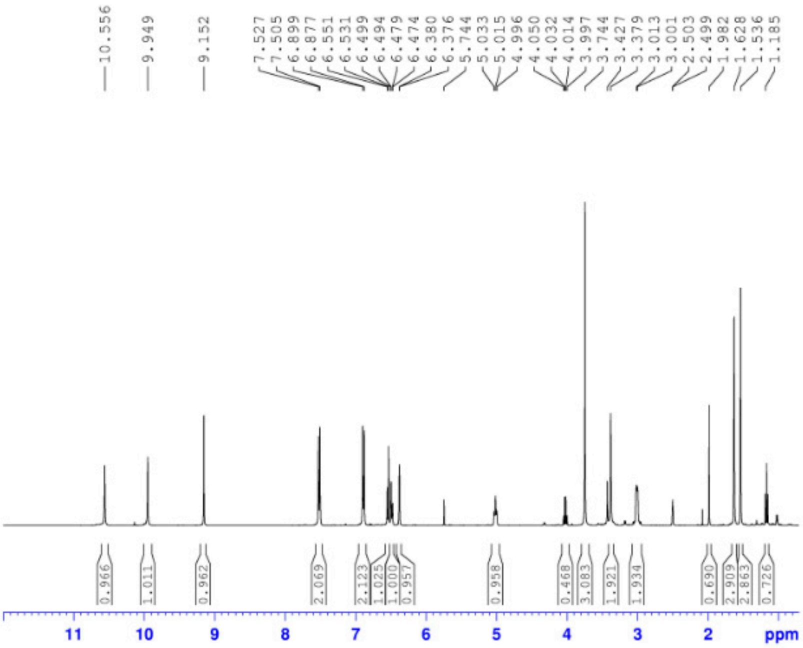

Figure S24. The ^{1}H-NMR (DMSO-d_6, 400 MHz) data of **4**.

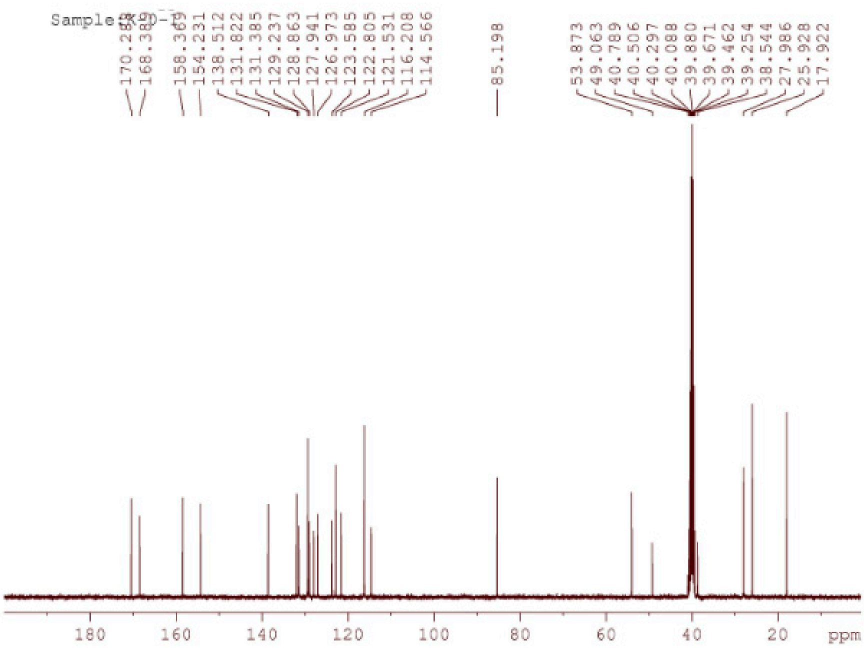

Figure S25. The ^{13}C-NMR (DMSO-d_6, 100 MHz) data of **4**.

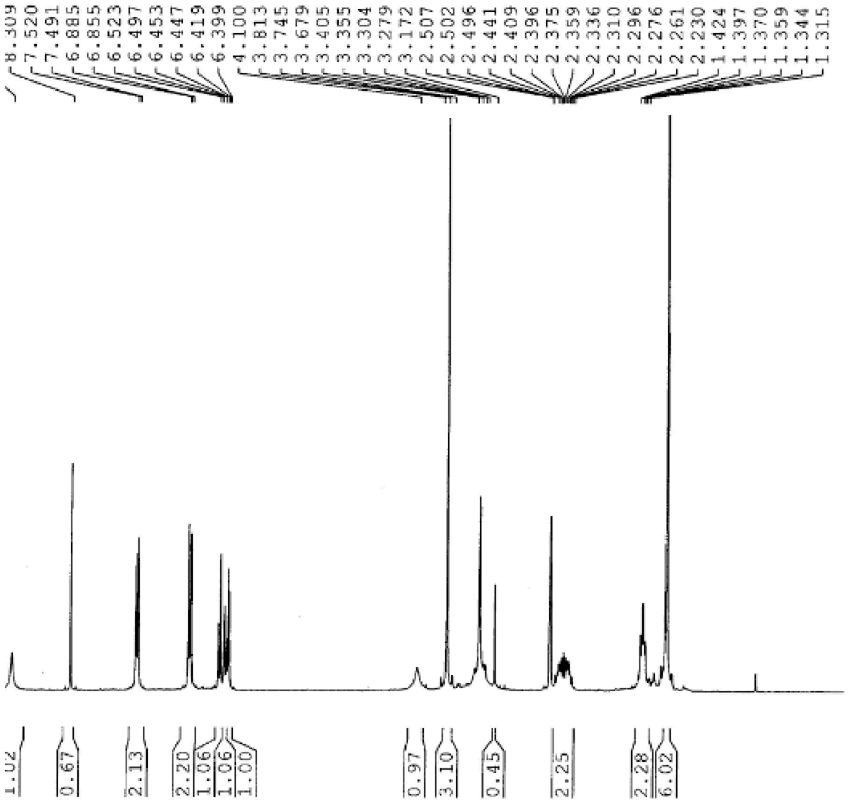

Figure S26. The ^{1}H-NMR (DMSO-d_6, 300 MHz) data of **5**.

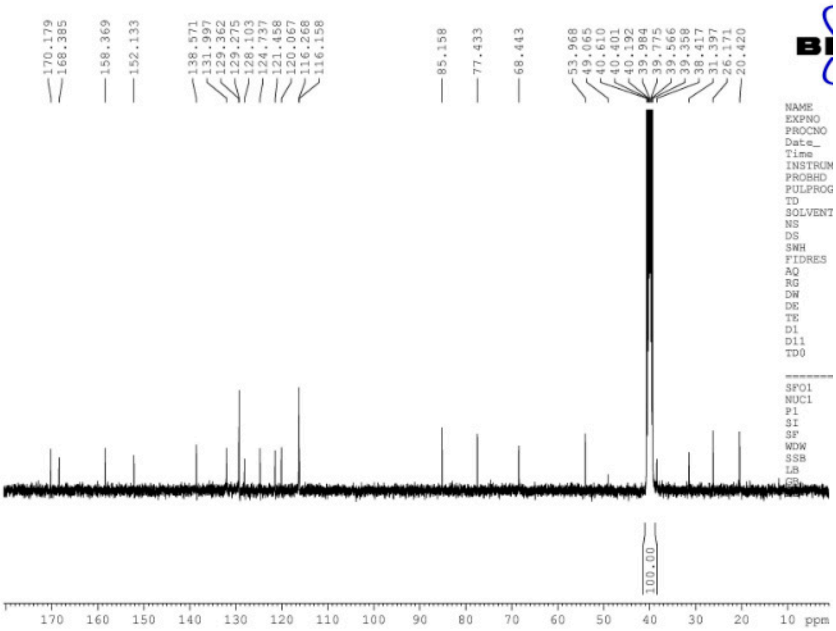

Figure S27. The ^{13}C-NMR (DMSO-d_6, 75 MHz) data of **5**.

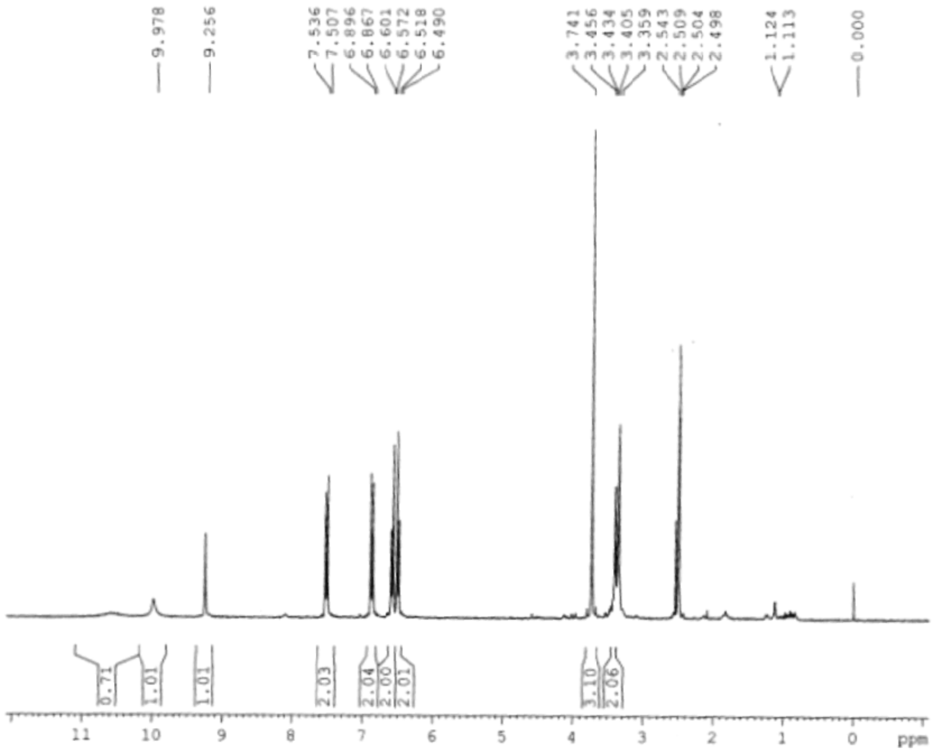

Figure S28. The ^{1}H-NMR (DMSO-d_6, 400 MHz) data of **6**.

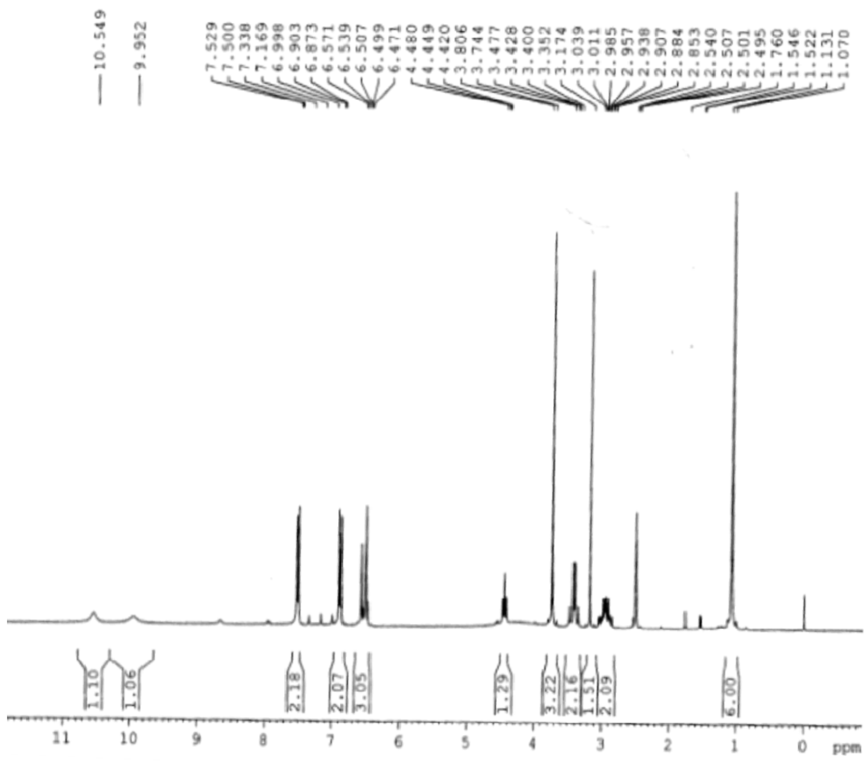

Figure S29. The ^{1}H-NMR (DMSO-d_6, 400 MHz) data of **7**.

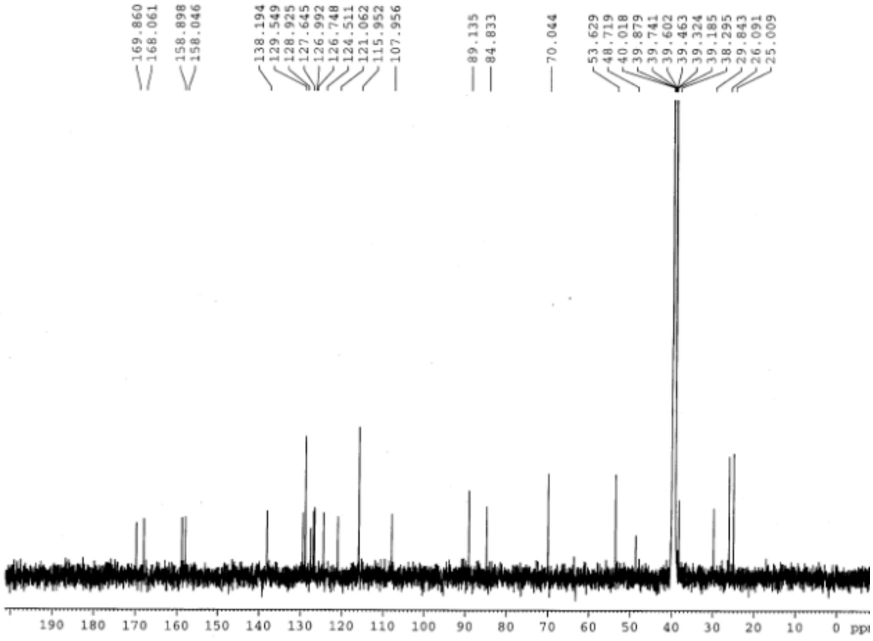

Figure S30. The ^{13}C-NMR (DMSO-d_6, 100 MHz) data of **7**.

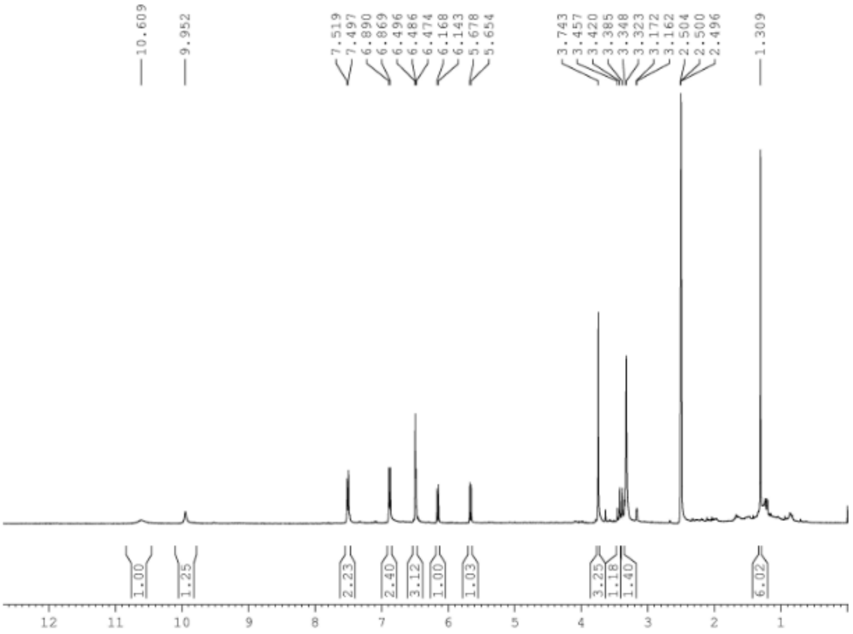

Figure S31. The ^{1}H-NMR (DMSO-d_6, 400 MHz) data of **8**.

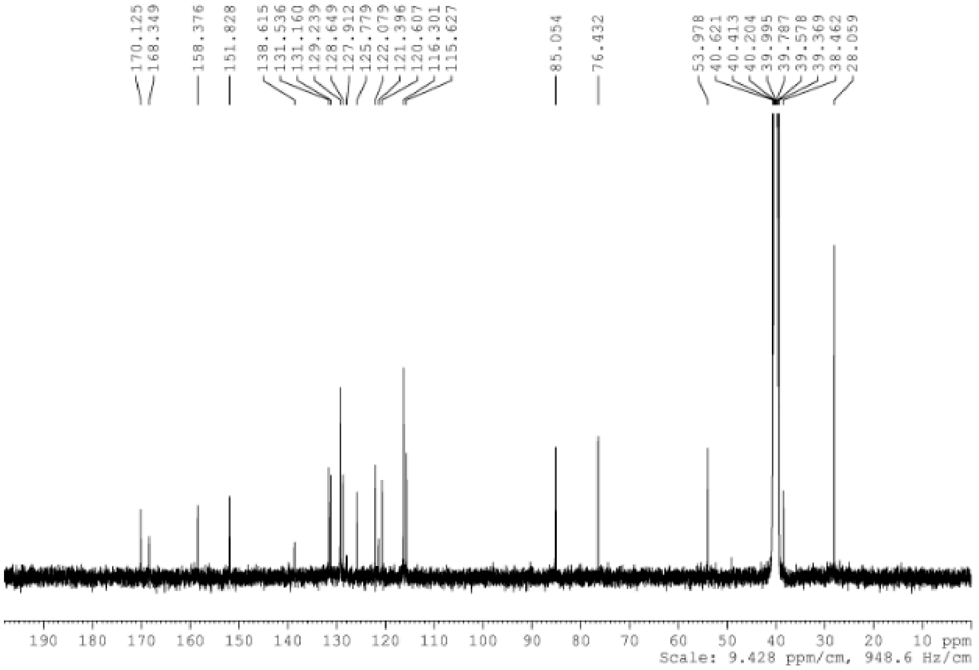

Figure S32. The ^{13}C-NMR (DMSO-d_6, 100 MHz) data of **8**.

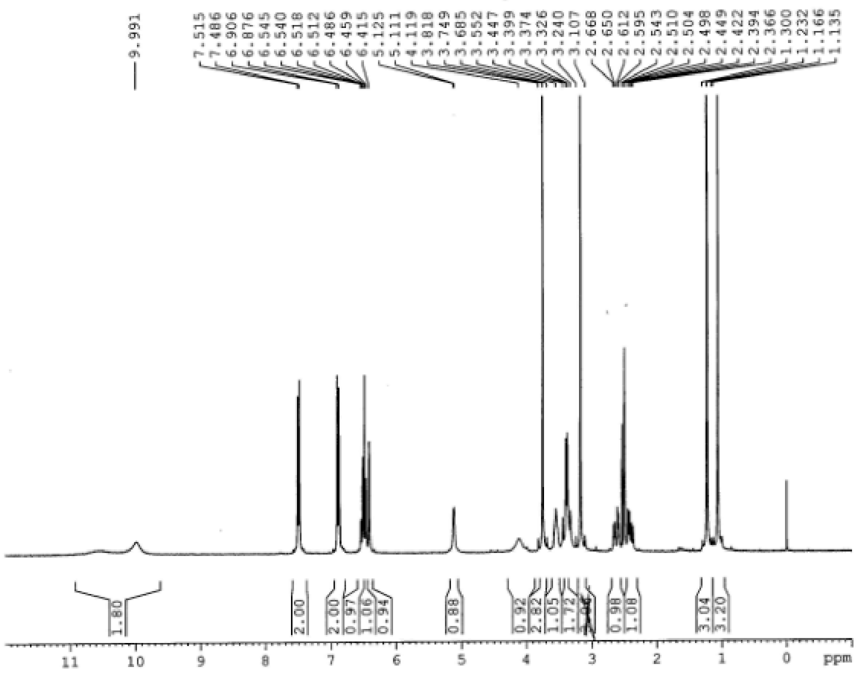

Figure S33. The ^{1}H-NMR (DMSO-d_6, 400 MHz) data of **9**.

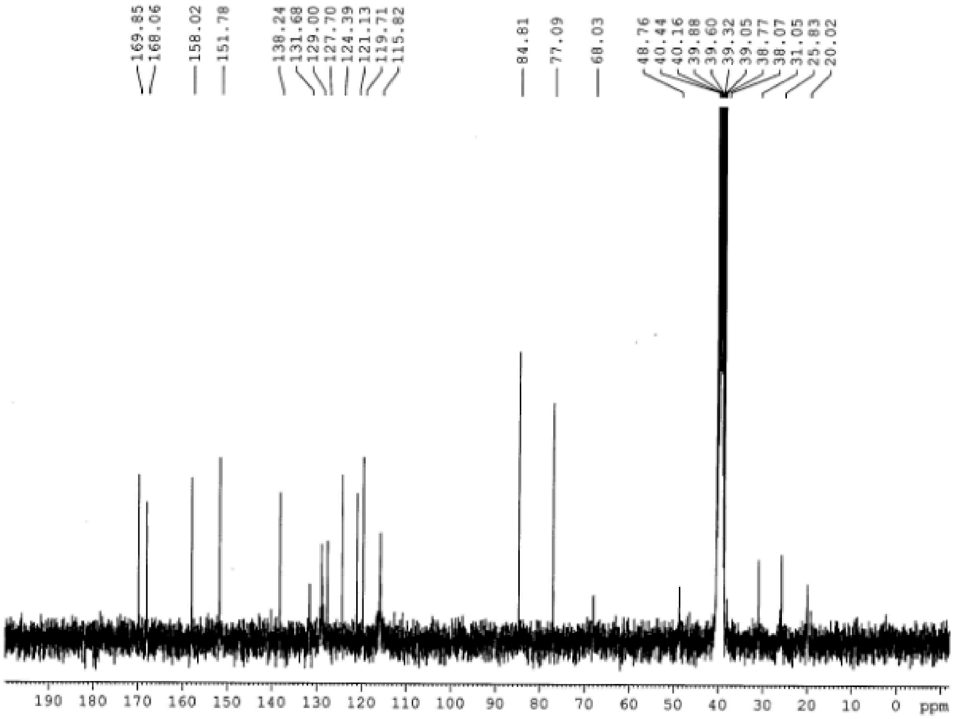

Figure S34. The ^{13}C-NMR (DMSO-d_6, 100 MHz) data of **9**.

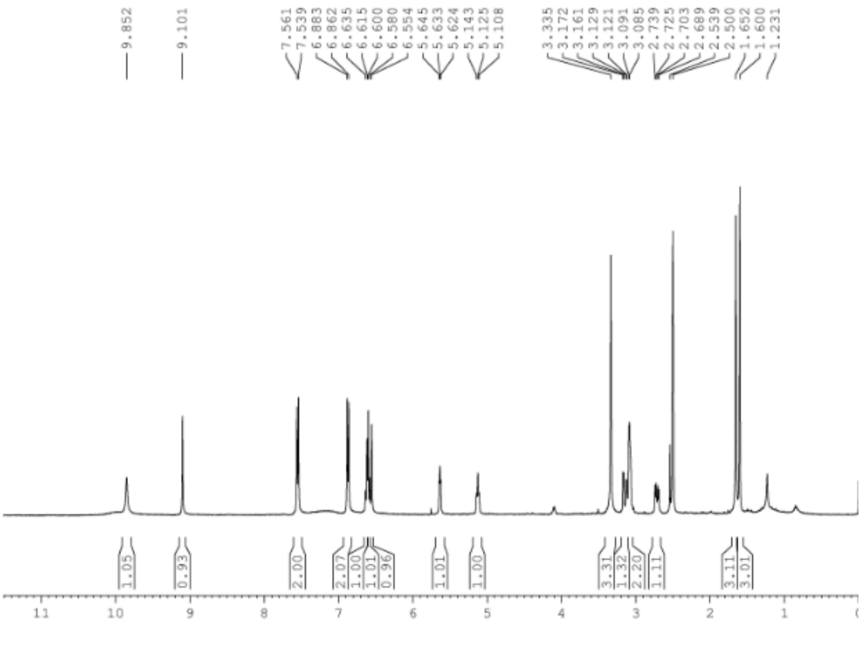

Figure S35. The ^{1}H-NMR (DMSO-d_6, 400 MHz) data of **10**.

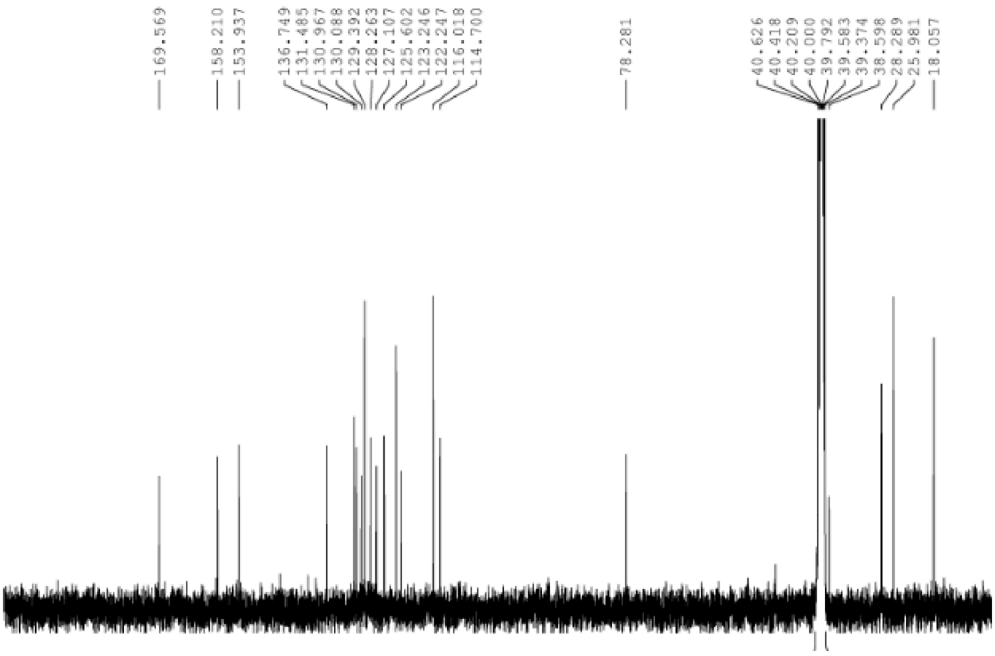

Figure S36. The ^{13}C-NMR (DMSO-d_6, 100 MHz) data of **10**.
